# Association Between Cardiometabolic Index and Blood Pressure: A Cross-Sectional Analysis of the NHANES 2015–2018 Data

**DOI:** 10.31083/RCM37359

**Published:** 2025-05-20

**Authors:** Lingyan He, Ling Sun, Haihua Pan, Changlin Zhai

**Affiliations:** ^1^Jiaxing University Master Degree Cultivation Base, Zhejiang Chinese Medical University, 310053 Hangzhou, Zhejiang, China; ^2^Department of Cardiology, The First Hospital of Jiaxing Affiliated Hospital of Jiaxing University, 314001 Jiaxing, Zhejiang, China

**Keywords:** cardiometabolic index (CMI), clinic blood pressure, cardiovascular risk, NHANES, multivariate regression analysis

## Abstract

**Background:**

Hypertension is a major risk factor for cardiovascular diseases (CVDs) and is closely related to metabolic abnormalities. The cardiometabolic index (CMI) integrates lipid profiles and anthropometric indicators, reflecting overall cardiometabolic health. However, the CMI and blood pressure (BP) relationship is poorly understood. Therefore, this study aimed to investigate the correlation between CMI and clinical BP and evaluate the potential of using this correlation as a cardiovascular risk indicator.

**Methods:**

National Health and Nutrition Examination Survey (NHANES) data from 2015 to 2018 were used to calculate the CMI based on the triglycerides to high-density lipoprotein cholesterol ratio and the waist-to-height ratio. The relationship between CMI and systolic blood pressure (SBP)/diastolic blood pressure (DBP) was analyzed using multivariate regression, threshold effect analysis, and subgroup analysis.

**Results:**

In this study cohort of 4240 participants, CMI positively correlated with SBP and DBP. After adjusting for age, gender, and race, the partial correlation for SBP was 0.56 (95% CI: 0.19–0.93; *p* < 0.01), while for DBP, it was 1.15 (95% CI: 0.60–1.71; *p* < 0.001). The threshold effect analysis revealed a positive association with SBP when the CMI was below 6.83 (β = 1.44, 95% CI: 0.64–2.24; *p* < 0.001) and a negative association when the CMI was above 6.83 (β = –1.52, 95% CI: –2.77– –0.28; *p* = 0.0123). For the DBP, a positive correlation was found when the CMI was below 2.81 (β = 1.45, 95% CI: 0.10–2.79; *p* = 0.0345), and a negative correlation when the CMI was above 2.81 (β = –1.92, 95% CI: –3.08– –0.77; *p* = 0.0012). A strong interaction was observed between the CMI and gender for the SBP (*p* = 0.0054) and a trend for the interaction between CMI and age for the DBP (*p* = 0.1667).

**Conclusions:**

This study found a significant positive correlation between the CMI and BP, with threshold effects supporting a non-linear relationship. The strong interaction between the CMI and gender for SBP suggests that the influence of the CMI on BP may be gender-dependent. These results highlight the importance of utilizing CMI in personalized cardiovascular risk stratification and underscore the relevance of considering patient factors such as gender in managing hypertension.

## 1. Introduction 

Hypertension is one of the most important risk factors for cardiovascular disease (CVD) worldwide, and as such it constitutes a serious threat to cardiovascular health [1]. Hypertension’s complications, including heart disease, stroke, and renal disease, were estimated by the WHO to account for over 9 million deaths annually [2]. As well as increasing cardiovascular events, hypertension has also been associated with dementia and cognitive decline [3]. Early identification and treatment of hypertension are therefore critical for the prevention of CVD and its reduction in mortality.

The cardiometabolic index (CMI) is a comprehensive indicator that is strongly associated with metabolic diseases such as diabetes and is also related to an increased risk of cardiovascular and cerebrovascular events, including stroke [4, 5]. The CMI offers a quantitative approach to the assessment of cardiometabolic risk by incorporating the ratios of waist circumference to height, and triglycerides to high-density lipoprotein cholesterol. A higher value of CMI indicates greater cardiometabolic risk, and detects normal-weight but metabolically abnormal individuals, i.e., metabolically obese normal weight (MONW) individuals [6, 7]. CMI provides useful data in the primary screening of high-risk patients, and in the clinical setting is mainly used to ascertain risk for metabolic syndrome, diabetes, and CVD [4, 8, 9].

Although previous studies have explored the relationship between cardiovascular risk and CMI [10, 11], the relationship between CMI and clinic blood pressure (BP) is uncertain. Clarification of this relationship should provide significant insights into the metabolic processes involved in the determination of BP, as well as new strategies for its management. The predictive value of CMI for cardiovascular events could also create new avenues for the prevention and treatment of CVD. In the present study, we therefore utilized the National Health and Nutrition Examination Survey (NHANES) database to investigate the correlation between CMI and clinical BP. Our aim was to provide a scientific foundation for the improvement of CVD prevention and control strategies.

## 2. Materials and Methods

### 2.1 Study Design

This cross-sectional analysis was conducted using data from the NHANES, a national, cross-sectional survey of the Centers for Disease Control and Prevention (CDC) to assess the health and nutritional status of adults and children in the United States. The research data were provided on the following website: https://wwwn.cdc.gov/nchs/nhanes/default.aspx. Consent was initially requested for all the anthropometric measurements and blood draws, and medical history was requested from all the participants.

### 2.2 Study Population and Exclusion Criteria

The initial population involved in this research included 19,226 participants of the NHANES database enrolled in 2015–2018. The following exclusion criteria were applied to attain the reliability and validity of our outcome:

① Excluding participants <18 years old (n = 346): Adolescents were excluded because they have significantly different metabolic profiles and mechanisms to control BP compared with adults. ② Exclusion of individuals who were taking antihypertensive medications (n = 285): The rationale for this exclusion is two-fold. First is the possibility of confounding effects on BP measurements. Antihypertensive medications directly lower BP through various mechanisms, such as reducing peripheral vascular resistance and modulating the renin-angiotensin-aldosterone system [12], which could mask the true relationship between CMI and BP. Second is the possibility of altered metabolic profiles due to medication use. The medications also affect lipid metabolism and insulin sensitivity [13] and hence may interfere with calculation and interpretation of the CMI. ③ Exclusion of missing data cases for clinic systolic blood pressure (SBP) and diastolic blood pressure (DBP) (n = 438): Subjects with missing BP data were excluded in order to ensure the validity and integrity of main outcome measures. ④ Exclude the cases with incomplete computation of the CMI (n = 13,917): Participants who had insufficient data to calculate the CMI (e.g., lacking triglycerides, high-density lipoprotein cholesterol (HDL-C), waist circumference, or height measurements) were excluded to ensure the guarantee of the consistency and validity of the CMI as a critical independent variable.

**Fig. 1.**
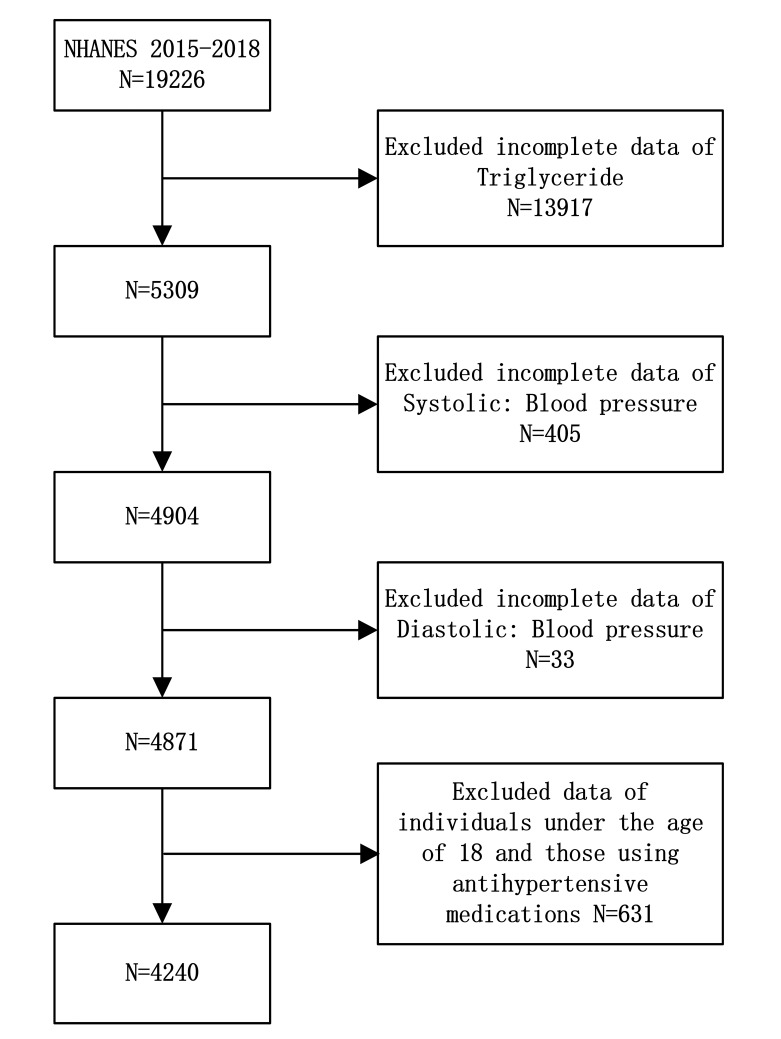
**Study flow chart**. NHANES, National Health and Nutrition Examination Survey.

After applying the exclusion criteria, the final population of the study comprised 4240 subjects. (Fig. 1).

### 2.3 Calculation of the Cardiometabolic Index

The CMI is an overall measure of metabolic risk as it includes measures of lipids and anthropometric markers of an individual’s cardiometabolic risk [4]. The four measures used in the current study to estimate CMI were triglycerides (TG), HDL-C, waist circumference, and height. With the high-density lipoprotein cholesterol-to-triglyceride ratio, CMI could be determined from the following formula: CMI = WHtR × (TG/HDL-C) [14, 15, 16], where WHtR is waist-to-height ratio (waist size to height ratio), TG is triglyceride level, and HDL-C is high-density lipoprotein cholesterol level. The CMI formula integrates the WHtR and TG/HDL-C ratio into a single index of not just the severity of obesity but also lipid abnormality, thus reflecting an overall determination of metabolic status of health.

### 2.4 Clinic Blood Pressure Measurement

The clinic BP measurements in the NHANES database conform to American Heart Association and American Society of Hypertension guidelines. The BP measurements are made in a quiet, temperature-controlled room, with the participants resting quietly for at least 5 minutes before measurement. SBP and DBP are measured using calibrated automated sphygmomanometers, with the results averaged over three measures.

### 2.5 Covariates

The covariates analysed included age, gender, race, family income-to-poverty ratio, body mass index (BMI), C-reactive protein levels, alcohol and smoking habits, diabetes status, and sleep disorders that may influence BP and the CMI. Data on these covariates were obtained from questionnaires, physical examinations, and laboratory tests.

### 2.6 Quality Control

Data were subjected to severe cleaning and quality control, including processing of missing data, screening and elimination of outliers, and checking measurement for errors. Poverty income ratio (PIR) data was missing for 467 cases, CRP data for 17 cases, alcohol use data for 754 cases, and smoking data for 2441 cases. To account for missing data, multiple imputation based on 5 replications and a chained equation approach method was adopted in the R MI procedure.

### 2.7 Statistical Analysis

Linear regression was used to assess the relationship between CMI and clinic BP. Modeling was performed under three different levels: non-adjusted model, Adjusted I model, and Adjusted II model. The Adjusted I model controlled for basic covariates such as age, gender, and race, while the Adjusted II model controlled for additional potential confounders, including alcohol and smoking habits, C-reactive protein (CRP) levels, diabetes status, and sleep disorders. Additionally, threshold effect analysis and interaction tests were conducted to explore the relationship between CMI and BP in different subgroups. Continuous variables are presented as mean ± standard deviation (Mean ± SD), and categorical variables are presented as frequencies (%). When comparing across three groups, continuous variables were analyzed using one-way analysis of variance (ANOVA), and categorical variables were analyzed using the Chi-square test. Normality tests were performed for all continuous variables using the Shapiro-Wilk test to confirm data distribution. All statistical analyses were performed using the EmpowerStats 4.2 (X&Y Solutions, Inc., Wilmington, Delaware, USA), with the significance level set at *p* < 0.05.

## 3. Results

### 3.1 Characteristics of the Study Participants

This study included a total of 4240 participants, with 47.8% being male and 52.2% being female. The average age was 48.85 ± 17.93 years. Baseline characteristics of the participants grouped according to tertiles of CMI are shown in Table 1 and include data on age, family income-to-poverty ratio (FAMILY.PIR), C-reactive protein (CRP), SBP and DBP. Both SBP and DBP were found to increase significantly with increasing CMI (*p* < 0.001).

**Table 1.** **Demographic characteristics of the study participants stratified according to CMI tertiles**.

Variable		Low CMI	Middle CMI	High CMI	*p*-value
		(0.19 ± 0.07)	(0.46 ± 0.10)	(1.38 ± 1.33)	
AGE, years		44.86 ± 19.30	50.27 ± 18.12	51.43 ± 16.38	<0.001
FAMILY.PIR		2.66 ± 1.57	2.47 ± 1.53	2.42 ± 1.49	<0.001
BMI, kg/m^2^		25.58 ± 5.60	29.96 ± 6.46	32.60 ± 6.88	<0.001
CRP, mg/L		2.97 ± 8.98	4.04 ± 5.71	5.13 ± 8.46	<0.001
SBP		120.55 ± 18.31	126.75 ± 20.00	127.88 ± 17.89	<0.001
DBP		68.36 ± 10.99	71.22 ± 12.22	72.22 ± 12.09	<0.001
GENDER					<0.001
	Male	580 (41.05%)	674 (47.70%)	830 (58.70%)	
	Female	833 (58.95%)	739 (52.30%)	584 (41.30%)	
ALCOHOL.USE					0.507
	Yes	216 (15.29%)	245 (17.34%)	245 (17.33%)	
	No	1197 (84.71%)	1168 (82.66%)	1169 (82.67%)	
SMOKING					0.925
	Every day	430 (30.43%)	421 (29.79%)	444 (31.40%)	
	Some days	143 (10.12%)	144 (10.19%)	143 (10.11%)	
	Not at all	840 (59.45%)	848 (60.01%)	827 (58.49%)	
RACE					<0.001
	Mexican American	143 (10.12%)	258 (18.26%)	284 (20.08%)	
	Other Hispanic	116 (8.21%)	169 (11.96%)	212 (14.99%)	
	Non-Hispanic White	463 (32.77%)	453 (32.06%)	521 (36.85%)	
	Non-Hispanic Black	422 (29.87%)	318 (22.51%)	155 (10.96%)	
	Non-Hispanic Asian	214 (15.15%)	146 (10.33%)	173 (12.23%)	
	Other Race-Including Multi-Racial	55 (3.89%)	69 (4.88%)	69 (4.88%)	
DIABETES					<0.001
	Yes	88 (6.23%)	208 (14.72%)	335 (23.69%)	
	No	1295 (91.65%)	1168 (82.66%)	1029 (72.77%)	
	Borderline	30 (2.12%)	37 (2.62%)	50 (3.54%)	
SLEEP.DISORDERS					<0.001
	Yes	309 (21.87%)	397 (28.10%)	452 (31.97%)	
	No	1104 (78.13%)	1016 (71.90%)	962 (68.03%)	

Mean ± SD for continuous variables; N (%) for categorical variables. CMI, cardiometabolic index; FAMILY.PIR, family income-to-poverty ratio; BMI, body mass index; CRP, C-reactive protein; SBP, systolic blood pressure; DBP, diastolic blood pressure.

### 3.2 Analysis of the Correlation Between CMI and Clinic BP

Table 2 shows the results of multivariate regression analysis of the relationship between CMI and BP. In the unadjusted model, CMI was positively correlated with both SBP (β = 1.07, 95% CI: 0.68–1.46, *p* < 0.001) and DBP (β = 1.63, 95% CI: 1.01–2.25, *p* < 0.001). In the Adjusted I model, the correlation between CMI and SBP was weaker but remained significant after controlling for factors such as age, gender, and race (β = 0.56, 95% CI: 0.19–0.93, *p* < 0.01), and similarly for DBP (β = 1.15, 95% CI: 0.60–1.71, *p* < 0.001). Following additional adjustment for potential confounders such as alcohol consumption, smoking, CRP levels, diabetes status, and sleep disorders in the Adjusted II model, the correlation between CMI and SBP weakened further but remained significant (β = 0.51, 95% CI: 0.13–0.89, *p* < 0.01), and likewise for DBP (β = 0.83, 95% CI: 0.27–1.40, *p* < 0.01).

**Table 2.** **Analysis of the correlation between CMI and clinic BP**.

		Non-adjusted	Adjusted I	Adjusted II
Y = SBP				
CMI		1.07 (0.68, 1.46) ^c^	0.56 (0.19, 0.93) ^b^	0.51 (0.13, 0.89) ^b^
CMI Tertile				
	Low	0	0	0
	Middle	2.86 (1.99, 3.73) ^c^	2.31 (1.48, 3.14) ^c^	2.24 (1.39, 3.09) ^c^
	High	3.85 (2.99, 4.72) ^c^	2.65 (1.79, 3.50) ^c^	2.65 (1.73, 3.56) ^c^
Y = DBP				
CMI		1.63 (1.01, 2.25) ^c^	1.15 (0.60, 1.71) ^c^	0.83 (0.27, 1.40) ^b^
CMI Tertile				
	Low	0	0	0
	Middle	6.20 (4.82, 7.58) ^c^	4.03 (2.79, 5.28) ^c^	3.52 (2.26, 4.79) ^c^
	High	7.34 (5.96, 8.72) ^c^	5.23 (3.94, 6.52) ^c^	4.44 (3.09, 5.79) ^c^

^b^*p* < 0.01; ^c^*p* < 0.001. Model 1: no covariates were adjusted. Model 2: age, gender, and race were adjusted. Model 3: age, gender, race, FAMILY.PIR, BMI, CRP, smoking, alcohol use, diabetes, sleep disorders were adjusted. BP, blood pressure.

### 3.3 Threshold Effect Analysis of CMI and Clinic BP

**Fig. 2.**
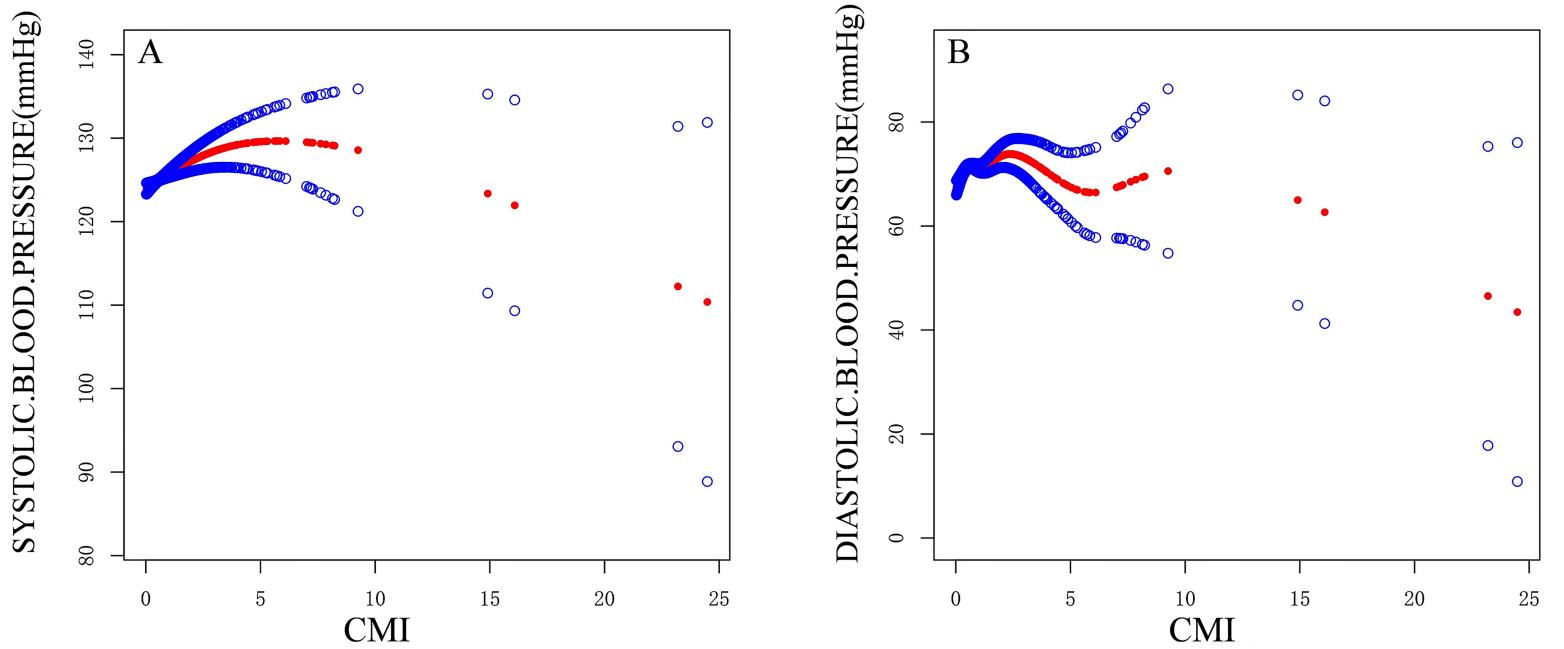
**Fitted curve of CMI and clinic BP**. (A) Fitted Curve of CMI and SBP. (B) Fitted Curve of CMI and DBP.

Fig. 2 and Table 3 present the results of the threshold effect analysis on the relationship between CMI and BP. A distinct inflection point (K = 6.83) was observed for SBP, below which CMI was positively correlated with SBP (β = 1.44, 95% CI: 0.64–2.24, *p* < 0.001). Above this inflection point, CMI was negatively correlated with SBP (β = –1.52, 95% CI: –2.77– –0.28, *p* = 0.0123). The inflection point for DBP was K = 2.81, below which CMI was positively correlated with DBP (β = 1.45, 95% CI: 0.10–2.79, *p* = 0.0345), and above which CMI was negatively correlated with DBP (β = –1.92, 95% CI: –3.08– –0.77, *p* = 0.0012).

**Table 3.** **Threshold effect analysis of the correlation between CMI and clinic BP**.

Outcome		SBP	DBP
Model I			
	Linear effect	0.44 (–0.13, 1.01)	–0.99 (–2.11, 0.14)
Model II			
	Inflection point (K)	6.83	2.81
	Effect below K (1)	1.44 (0.64, 2.24) ^c^	1.45 (0.10, 2.79) ^a^
	Effect above K (2)	–1.52 (–2.77, –0.28) ^a^	–1.92 (–3.08, –0.77) ^b^
	Difference between effects 2 and 1	–2.96 (–4.64, –1.29) ^c^	–3.37 (–4.39, –2.35) ^c^
	Predicted value at inflection point	145.43 (140.23, 150.63)	76.85 (75.38, 78.31)
	Log–likelihood ratio test	<0.001	<0.001

^a^*p* < 0.05; ^b^*p* < 0.01; ^c^*p* < 0.001. Adjustment was made for age, gender, race, FAMILY.PIR, BMI, CRP, smoking, alcohol use, diabetes, sleep disorders.

### 3.4 Sensitivity Analysis

Table 4 presents the results of the sensitivity analysis, revealing that the results from the complete case analysis were consistent with those from the multiple imputation analysis. In the Adjusted II model, the complete case analysis showed a significant positive association between CMI and SBP (β = 0.48, 95% CI: 0.17–0.78, *p* = 0.0023), which was similar to the results from the multiple imputation analysis (β = 0.51, 95% CI: 0.13–0.89, *p* < 0.01). Similarly, for DBP, the complete case analysis results (β = 0.24, 95% CI: 0.04–0.45, *p* = 0.0214) were consistent with the multiple imputation analysis results (β = 0.83, 95% CI: 0.27–1.40, *p* < 0.01). These consistent findings suggest that the missing data on smoking history did not introduce significant bias or alter the primary results.

**Table 4.** **Sensitivity analysis: comparison of results from complete case analysis and multiple imputation**.

Outcome	Model	Complete Case Analysis (n = 1799)	Multiple Imputation (n = 4240)
SBP	Unadjusted	β = 0.87 (0.52, 1.21) ^c^	β = 1.07 (0.68, 1.46) ^c^
	Adjusted I	β = 0.44 (0.13, 0.75) ^b^	β = 0.56 (0.19, 0.93) ^b^
	Adjusted II	β = 0.48 (0.17, 0.78) ^b^	β = 0.51 (0.13, 0.89) ^b^
DBP	Unadjusted	β = 0.46 (0.25, 0.68) ^c^	β = 1.63 (1.01, 2.25) ^c^
	Adjusted I	β = 0.43 (0.21, 0.64) ^c^	β = 1.15 (0.60, 1.71) ^c^
	Adjusted II	β = 0.24 (0.04, 0.45) ^a^	β = 0.83 (0.27, 1.40) ^b^

^a^*p* < 0.05; ^b^*p* < 0.01; ^c^*p* < 0.001. Adjustment was made for age, gender, race, FAMILY.PIR, BMI, CRP, smoking, alcohol use, diabetes, sleep disorders.

### 3.5 Interaction Analysis

Table 5 presents results of the interaction tests between CMI and factors such as gender and age. A significant interaction was observed between CMI and gender in SBP (*p* = 0.0054), suggesting the association between CMI and SBP differs between males and females. Specifically, the effect of CMI on SBP was stronger in females (β = 5.74, 95% CI: 4.17–7.31, *p* < 0.001) compared to males (β = 0.51, 95% CI: –0.13–1.14).

**Table 5.** **Interaction analysis of the correlation between CMI and clinic BP due to related factors**.

Variables		N	SBP	*p* interaction	DBP	*p* interaction
GENDER				0.0054		0.0936
	Male	2084	0.51 (–0.13, 1.14)		0.75 (0.31, 1.19) ^c^	
	Female	2156	5.74 (4.17, 7.31) ^c^		1.56 (0.66, 2.45) ^c^	
AGE, years				0.8865		0.1667
	≤35	1153	3.03 (2.02, 4.04) ^c^		2.63 (1.68, 3.58) ^c^	
	>35, ≤60	1663	1.16 (0.39, 1.93) ^b^		0.74 (0.25, 1.24) ^b^	
	>60	1424	0.23 (–0.99, 1.45)		–0.15 (–0.87, 0.57)	
CRP, mg/L				0.4026		0.2285
	<10	3887	1.58 (0.95, 2.21) ^c^		1.12 (0.73, 1.51) ^c^	
	≥10	353	2.23 (–0.92, 5.39)		–0.09 (–2.15, 1.98)	
SLEEP.DISORDERS				0.9652		0.9177
	Yes	1158	1.22 (0.01, 2.42) ^a^		0.91 (0.12, 1.70) ^a^	
	No	3082	1.71 (0.99, 2.43) ^c^		1.10 (0.66, 1.55) ^c^	
DIABETES				0.9369		0.3462
	Yes	631	–0.77 (–2.16, 0.62)		0.46 (–0.39, 1.32)	
	No	3492	1.68 (1.00, 2.37) ^c^		1.30 (0.86, 1.74) ^c^	
	Borderline	117	–1.73 (–10.05, 6.59)		–0.73 (–5.20, 3.74)	

^a^*p* < 0.05; ^b^*p* < 0.01; ^c^*p* < 0.001.

## 4. Discussion

This study used the NHANES database to explore the correlation between CMI and clinic BP. Multivariate regression analysis found that CMI was positively correlated with both SBP and DBP. Moreover, a consistent relationship remained across different age, gender, and race subgroups. The significant association observed between CMI and BP possibly reflects common metabolic pathways and biological mechanisms.

Threshold effect analysis revealed a non-linear relationship between CMI and BP, with critical thresholds identified for CMI. For SBP, an inflection point was observed at a CMI value of 6.83, indicating that CMI increases when SBP is below this threshold, but decreases above it. For DBP, an inflection point was observed at a CMI value of 2.81, and a similar non-linear relationship was observed between CMI and DBP. The results suggest that biological thresholds may play a role in modulating the relationship between CMI and BP. This is likely to be related to the complex mechanisms of BP regulation, including changes in sensitivity to metabolic factors across different metabolic states.

The non-linear relationship observed here may be attributed to several underlying mechanisms. First, when CMI is at a lower level, the metabolic abnormalities reflected by CMI (e.g., elevated triglycerides, low HDL-C, and increased waist-to-height ratio) primarily influence BP through inflammation [17] and endothelial dysfunction [18], resulting in a positive correlation between CMI and BP. Specifically, these metabolic abnormalities trigger systemic inflammatory responses that increase pro-inflammatory cytokines (e.g., tumor necrosis factor‌ (TNF)-α, interleukin (IL)-6) while reducing anti-inflammatory adipokines (e.g., adiponectin). The inflammatory markers impair endothelial function by reducing nitric oxide (NO) bioavailability [19], leading to decreased vasodilation and increased vascular stiffness, thereby raising BP. Additionally, an increased waist-to-height ratio is indicative of abdominal obesity, which is closely associated with insulin resistance [20], further exacerbating endothelial dysfunction and promoting hypertension.

However, when CMI exceeds certain thresholds, the relationship between CMI and BP changes significantly. The present study found distinct inflection points of 6.83 for SBP and 2.81 for DBP. This phenomenon may be related to the complex mechanisms of BP regulation. When CMI exceeds 6.83, it becomes negatively correlated with SBP, possibly because under extreme metabolic abnormalities (e.g., severe obesity or insulin resistance), the body activates a series of counter-mechanisms that prevent further increases in BP. For example, the renin-angiotensin-aldosterone system (RAAS) may be activated, but as metabolic abnormalities worsen, the feedback regulation mechanisms for RAAS may change, thereby weakening its regulatory effect on BP and even leading to paradoxical responses [21]. Additionally, the sympathetic nervous system (SNS) is often overactivated in metabolic syndrome, which may lead to receptor fatigue or desensitization when metabolic abnormalities reach a certain level [22], thus reducing its pressor effects.

When CMI exceeds 2.81, it also becomes negatively correlated with DBP. This may be related to further deterioration of endothelial function. When CMI is low, endothelial dysfunction is mainly manifested as reduced NO bioavailability, leading to decreased vasodilation. However, as CMI increases, endothelial dysfunction may worsen [18], reducing the responsiveness of blood vessels to vasodilatory signals and even offsetting the effects of vasoconstriction. Additionally, as metabolic abnormalities worsen, adipose tissue may secrete more anti-inflammatory factors (e.g., adiponectin) [23], which may improve endothelial function and reduce BP by reducing oxidative stress.

The different responses of SBP and DBP to changes in CMI may also be due to their different physiological mechanisms in relation to the formation and regulation of BP. SBP mainly reflects the peak BP during cardiac contraction, whereas DBP mainly reflects the maintenance of BP during cardiac relaxation. Therefore, SBP and DBP may have different sensitivities to metabolic abnormalities, leading to different correlations when CMI exceeds certain thresholds.

Interaction analysis revealed a significant interaction between CMI and gender for SBP (*p* = 0.0054), indicating the association between CMI and SBP differs between males and females. Specifically, the effect of CMI on SBP was stronger in females (β = 5.74, 95% CI: 4.17–7.31, *p* < 0.001) than in males (β = 0.51, 95% CI: –0.13–1.14), suggesting that gender plays a crucial role in modulating the relationship between CMI and SBP. The stronger association in females may be related to differences in cardiovascular physiology and metabolic characteristics, such as the protective effects of estrogen before menopause [24], and differences in lipid metabolism and insulin sensitivity [25, 26]. Our analysis did not reveal a significant interaction between CMI and age for SBP, indicating the relationship between CMI and BP was consistent across different age groups. However, age-related physiological changes such as increased vascular stiffness and decreased kidney function may still influence BP regulation [27, 28, 29].

Several studies have investigated the relationship between CMI and BP across different regions and populations. A study comprising 11,400 adults in China reported a significant positive association between CMI and hypertension [11]. Moreover, for each one standard deviation increase in CMI, the risk of hypertension increased by 35.6% in women and 31% in men. Another study of 15,453 participants in Japan revealed a nonlinear relationship between CMI and the risk of hypertension and other metabolic diseases [30]. The risk was significantly increased when CMI was low (<1.01), but the association weakened when CMI was high. A study based on adolescents found that CMI can effectively predict hypertension, with an AUC value from receiver operating characteristic (ROC) curve analysis of 0.710, indicating an acceptable degree of accuracy in screening for hypertension risk [10]. These studies vary in design and methodology, with some having cross-sectional designs and others being longitudinal cohort studies. Although most previous study results are consistent with our findings, some studies did not find significant associations, possibly due to differences in genetic background, lifestyle, dietary habits, and methodological differences in BP measurement. The methodological advantage of the current study lies in its use of the NHANES database, which is a representative national sample and thus more broadly generalizable. Additionally, our study revealed nonlinear characteristics and specific thresholds of the relationship between CMI and BP through threshold effect analysis. This offers new support for the clinical application of CMI, while contributing to a more precise assessment of individual cardiovascular risk.

As a comprehensive indicator of metabolic health, CMI may be related to BP through various biological pathways. Firstly, the ratio of triglycerides to high-density lipoprotein cholesterol in CMI reflects lipid abnormalities, which have been associated with endothelial dysfunction and vascular inflammation [31]. Endothelial dysfunction leads to a decrease in the vascular dilation capacity, thereby increasing the risk of cardiovascular events. Secondly, the waist-to-height ratio in CMI indicates abdominal obesity, a key component of metabolic syndrome, and is associated with insulin resistance and chronic inflammatory states [32, 33]. Furthermore, CMI is closely associated with the progression of vascular atherosclerosis. Studies have shown that the increase in CMI not only reflects metabolic abnormalities but may also accelerate the progression of atherosclerosis by promoting endothelial dysfunction [34] and inflammatory responses [35]. Wakabayashi *et al*. [6] further confirmed that CMI is significantly associated with the progression of atherosclerosis in patients with peripheral arterial disease. Therefore, CMI may serve as a potential indicator for assessing the risk of atherosclerosis. These pathophysiological processes collectively promote an elevated BP and increased cardiovascular risk.

The strengths of this study lie in its large sample size and national representativeness associated with the NHANES database, thereby enhancing the generalizability of our findings. Additionally, we employed multivariate regression analysis to control for several potential confounding factors, adding strength to the reliability of the results. However, there are also some limitations to this study. First, due to the cross-sectional nature of the NHANES data, causality from CMI to BP is not possible to determine. Second, although we controlled for different covariates, there could still be some unmeasured confounders such as genetic factors and psychosocial traits that could potentially affect the relationship between CMI and BP. Last but not least, although the computation formula for CMI is simple and easy to implement, application of it in diverse populations still needs further validation. It is also noteworthy that 2441 had missing information on smoking history. This may have introduced some bias and limited the generalizability of our findings, as smoking is a confirmed risk factor for hypertension and cardiovascular diseases. Future studies need to be geared towards addressing this shortcoming through the gathering of more detailed data on smoking practices.

Finally, this study reports novel evidence for the relationship between CMI and clinic BP and the potential of CMI for cardiovascular risk stratification. Even though our observations are limited by their nature, they provide the foundation for subsequent studies that might have important clinical implications for managing hypertension and other related disorders.

## 5. Conclusions

The CMI was found to be highly and positively correlated with clinic BP in the general United States population, which indicates its potential utility as a valuable predictor of cardiovascular risk. The correlation is particularly valuable in the identification of high-risk patients for hypertension and related cardiovascular complications. CMI follow-up can further elucidate how the BP of an individual is related to his or her metabolic health.

The threshold effects revealed in this research indicated substantial non-linear correlations between CMI and BP, and the influence of CMI on BP based on whether CMI was greater or less than specific thresholds. These data suggest that the treatment of hypertension should be tailored to an individual’s CMI score. For instance, when CMI is subthreshold, it should be addressed to manage other cardiovascular risk factors, whereas when CMI is over the threshold, intensified BP surveillance and treatment are appropriate.

Significant gender interactions with CMI for SBP emphasize the contribution of individual factors to the modification of the CMI-BP relationship. These interactions indicate that the association between CMI and BP is not uniform across the population, and can be influenced by factors such as gender. Therefore, a multi-factorial comprehensive analysis involving CMI and demographic and clinical factors is essential for identifying high-risk subgroups accurately and guiding targeted interventions.

## Data Availability

Publicly available datasets were analyzed in this study. These data can be found at: https://www.cdc.gov/nchs/nhanes/about/erb.html.

## Abbreviations

CMI, cardiometabolic index; NHANES, National Health and Nutrition Examination Survey; SBP, systolic blood pressure; DBP, diastolic blood pressure; CVDs, cardiovascular diseases; TG, triglycerides; HDL-C, high-density lipoprotein cholesterol; WHtR, waist-to-height ratio; BMI, body mass index; CRP, C-reactive protein; PIR, poverty income ratio; ROC, receiver operating characteristic; RAAS, renin-angiotensin-aldosterone system; SNS, sympathetic nervous system.
